# Evaluation of Biocontrol Measures to Reduce Bacterial Load and Healthcare-Associated Infections

**DOI:** 10.3390/microorganisms13081923

**Published:** 2025-08-18

**Authors:** Anna Vareschi, Salvatore Calogero Gaglio, Kevin Dervishi, Arianna Minoia, Giorgia Zanella, Lorenzo Lucchi, Elena Serena, Concepcion Jimenez-Lopez, Francesca Cristiana Piritore, Mirko Meneghel, Donato Zipeto, Diana Madalina Gaboreanu, Ilda Czobor Barbu, Mariana Carmen Chifiriuc, Luca Piubello Orsini, Stefano Landi, Chiara Leardini, Massimiliano Perduca, Luca Dalle Carbonare, Maria Teresa Valenti

**Affiliations:** 1 Department of Engineering for the Innovation Medicine, University of Verona, 37100 Verona, Italy; anna.vareschi@univr.it (A.V.); arianna.minoia@univr.it (A.M.); luca.dallecarbonare@univr.it (L.D.C.); 2 Department of Neurosciences, Biomedicine and Movement Sciences, University of Verona, 37100 Verona, Italy; salvatorecalogero.gaglio@univr.it (S.C.G.); kevin.dervishi@univr.it (K.D.); francescacristiana.piritore@univr.it (F.C.P.); mirko.meneghel@univr.it (M.M.); donato.zipeto@univr.it (D.Z.); 3 Department of Biotechnology, University of Verona, 37134 Verona, Italy; giorgia.zanella@univr.it; 4 STUDIO ASA, 31020 Villorba, Italy; l.lucchi@asalab.it (L.L.); e.serena@asalab.it (E.S.); 5 Department of Microbiology, University of Granada, 18071 Granada, Spain; cjl@ugr.es; 6 Department of Botany and Microbiology, Faculty of Biology, University of Bucharest, 030018 Bucharest, Romania; m.gaboreanu@bio.unibuc.ro (D.M.G.); ilda.barbu@bio.unibuc.ro (I.C.B.); carmen.chifiriuc@bio.unibuc.ro (M.C.C.); 7 Department of Management, University of Verona, Via Cantarane 24, 37129 Verona, Italy; luca.piubelloorsini@univr.it (L.P.O.); stefano.landi@univr.it (S.L.); chiara.leardini@univr.it (C.L.)

**Keywords:** hospital-acquired infections (HAIs), thermal disinfection, methyl ester sulfonates, antibacterial nanoparticles, wheelchair cover

## Abstract

Hospital-acquired infections (HAIs) remain a major clinical and economic burden, with pathogens such as Escherichia coli contributing to high rates of morbidity and mortality. Traditional manual disinfection methods are often insufficient, particularly in high-risk hospital environments. In this study, we investigated innovative strategies to enhance surface decontamination and reduce infection risk. First, we assessed the efficacy of the SMEG BPW1260 bedpan washer-disinfector, a thermal disinfection system for human waste containers. Our results demonstrated a reduction in *Clostridium difficile* and *Escherichia coli* contamination by >99.9% (>3 log reduction), as measured by colony-forming units (CFU) before and after treatment. Molecular techniques, including spectrophotometry, cell counting, and quantitative PCR (qPCR) for DNA quantification, confirmed reduction in bacterial contamination. Specifically, *Clostridium difficile* showed a reduction of approximately 89% in both optical density (OD) and cell count (cells/mL). In the case of *Escherichia coli*, a reduction of around 82% in OD was observed, with an even more pronounced decrease in cell count, reaching approximately 99.3%. For both bacteria, DNA quantification by qPCR was below detectable limits. Furthermore, we optimized the energy efficiency of the disinfection cycle, achieving a 45% reduction in power consumption compared to standard protocols without compromising antimicrobial efficacy. Secondly, we developed a sustainable cleaning solution based on methyl ester sulfonate surfactants derived from waste cooking oil. The detergent’s antibacterial activity was tested on contaminated surfaces and further enhanced through the incorporation of nanoassemblies composed of silver, electrostatically bound either to biomimetic magnetic nanoparticles or to conventional magnetic nanoparticles. Washing with the detergent alone effectively eliminated detectable contamination, while the addition of nanoparticles inhibited bacterial regrowth. Antimicrobial testing against *E. coli* revealed that the nanoparticle-enriched formulations reduced the average MIC values by approximately 50%, with MIC_50_ values around 0.03–0.06 mg/mL and MIC_90_ values between 0.06 and 0.12 mg/mL, indicating improved inhibitory efficacy. Finally, recognizing the infection risks associated with intra-hospital transport, we tested the SAFE-HUG Wheelchair Cover, a disposable non-woven barrier designed to reduce patient exposure to contaminated wheelchair surfaces. Use of the cover resulted in a 3.3 log reduction in surface contamination, based on viable cell counts. Optical density and bacterial DNA were undetectable in all covered samples at both 1 and 24 h, confirming the strong barrier effect. Together, these approaches—thermal no-touch disinfection, eco-friendly detergent boosted with nanoparticles, and protective transport barriers—respond to the urgent need for effective, sustainable infection control methods in healthcare settings. Our findings demonstrate the potential of these systems to counteract microbial contamination while minimizing environmental impact, offering promising solutions for the future of infection prevention in healthcare settings.

## 1. Introduction

Hospital-acquired infections (HAIs), also known as healthcare-associated infections (HCAIs), represent a major clinical and economic burden for healthcare systems worldwide [1]. These infections are defined as infections that are not present or incubating at the time of hospital admission but develop at least 48 h after admission [2]. HAIs affect millions of patients annually and are associated with increased morbidity, mortality, prolonged hospital stays, and higher healthcare costs. They are a leading cause of morbidity and mortality, with sepsis and sepsis-related deaths being primarily caused by diarrheal diseases (9.2 to 15 million cases annually) and lower respiratory infections (1.8 to 2.8 million cases annually, according to 2017 reports) [2]. Common pathogens responsible for HAIs include Staphylococcus aureus (including methicillin-resistant S. aureus, MRSA), Clostridium difficile, Escherichia coli, Pseudomonas aeruginosa, Klebsiella pneumoniae, various multidrug-resistant organisms (MDROs), as well as fungal and viral agents frequently implicated in HAIs [3]. These pathogens are often implicated in bloodstream infections, surgical site infections, urinary tract infections, pneumonia (particularly ventilator-associated pneumonia), and gastrointestinal infections [3]. The global distribution of HAIs varies, with higher incidence rates generally reported in low- and middle-income countries due to differences in healthcare infrastructure, infection control practices, and resource availability [4]. According to the World Health Organization (WHO), approximately 7% of hospitalized patients in developed countries and 10% or more in developing countries acquire at least one HAI during their hospital stay [5]. However, prevalence rates vary considerably between regions. For example, in Africa, pooled HAI prevalence estimates differ markedly by subregion: West Africa reports a prevalence of approximately 15.5%, East Africa shows an even higher rate around 19.7%, Central Africa about 10.3%, while Southern Africa has a lower prevalence near 6.5% [5]. These data highlight substantial regional disparities within developing countries, reflecting differences in healthcare infrastructure, infection prevention practices, and resource availability. The increasing prevalence of multidrug-resistant pathogens complicates treatment and highlights the urgent need for improved infection prevention and control strategies [6,7].

In healthcare settings, pathogens can be transmitted via direct contact with contaminated surfaces or through healthcare personnel [8]. Hospitals often rely on chemical disinfectants for environmental cleaning; however, numerous studies have shown that manual cleaning alone is insufficient to reduce contamination to safe levels [9]. *Escherichia coli* (*E. coli*) causes numerous types of infections, such as bloodstream infections (bacteremia), wound infections, infections of the gastrointestinal tract and urinary tract infections [10]. Urinary tract infections (UTIs) pose a significant challenge in healthcare settings [11]. UTIs arise from microbial colonization in the urinary tract and can lead to severe complications, such as pyelonephritis, cystitis, urethritis, epididymitis, and prostatitis, potentially progressing to bloodstream infections [12]. The most common causative agent, uropathogenic *E. coli*, is responsible for approximately 75% of uncomplicated UTIs and 65% of complicated ones [13]. The increasing prevalence of antimicrobial resistance among uropathogens further exacerbates the clinical and economic burden of UTIs [14]. In addition, *Clostridium difficile* (*C. difficile*) causes healthcare-associated infectious diarrhea, with an increasing number of community-acquired cases of colitis [15]. This Gram-positive, obligate anaerobic, spore-forming bacterium can survive for extended periods on contaminated surfaces, facilitating transmission through both symptomatic and asymptomatic carriers [15].

Infection control relies on strict cleaning protocols that, although crucial, still face major challenges that need to be addressed. On one hand, traditional cleaning methods in healthcare settings often rely on the repeated use of chemical disinfectants—such as quaternary ammonium compounds, chlorine-based agents (e.g., sodium hypochlorite), and alcohols—which can contribute to environmental pollution and the development of antimicrobial resistance [16,17]. Also, healthcare environments, particularly those involving vulnerable or mobility-impaired patients, face persistent challenges in maintaining effective and sustainable hygiene standards. Additionally, there is a growing concern about cross-contamination risks in shared medical equipment and spaces, especially in the context of pandemics and multidrug-resistant organisms. These issues highlight the urgent need for innovative, efficient, and environmentally responsible disinfection strategies.

In particular, healthcare facilities are increasingly adopting automated, no-touch disinfection technologies, such as bedpan washer-disinfectors (BWDs). These devices streamline the cleaning and decontamination of human waste containers, such as bedpans and urinals, directly in the ward, thus eliminating the need for the involvement of a central processing department (CPD) [18]. However, despite their widespread use, a validation using biological indicators is not required in the European Standard EN ISO 15883-3 [19,20,21], and, even if performed, conventional culture-mediated microbiological methods for pathogen identification are often time-consuming and unable to detect non-culturable microorganisms [22]. As such, new alternatives to improve infection control need to be studied, including the validation of already known disinfectants, the characterization of new ones, the implementation of novel strategies, and/or the development of easier and faster protocols for bacterial detection. The present paper addresses some of these concerns by studying three approaches.

Firstly, we investigated the efficacy of a bedpan washer (used in thermal disinfection), in reducing contaminants commonly found on hospital pans, specifically bacteria such as Clostridium species and *E. coli*. In addition to evaluating the system’s ability to reduce the bacterial load, we also investigated its sustainability by assessing energy consumption, aiming to determine whether effective disinfection could be achieved with an optimized energy-efficiency protocol. Secondly, also in line with the growing focus on circular economy principles, we addressed the environmental and sustainability challenges posed by traditional cleaning methods. These often rely on high resource consumption and the repeated use of chemical agents, contributing to environmental pollution and antimicrobial resistance [23,24]. To counteract these issues, we developed an innovative cleaning system that minimizes resource use and waste production, while maintaining high disinfection efficacy. Specifically, we formulated a detergent using methyl ester sulfonate surfactants derived from waste cooking oil, and its effectiveness in eliminating bacteria from surfaces was evaluated by rinsing contaminated surfaces and measuring bacterial reduction. Furthermore, we explored the integration of advanced technologies —such as the incorporation of nanoassemblies, based on silver (Ag) alternatively bound to biomimetic magnetic nanoparticles (BMNPs) or magnetic nanoparticles (MNPs)—able to release Ag ions together with the formulated detergent, to enhance its bactericidal properties, achieving a more efficient bacterial eradication. These combined efforts address the increasing demand for sustainable and effective decontamination strategies, paving the way for future innovations that balance microbial control with environmental sustainability. Thirdly, we propose a new alternative to improve infection control in wheelchairs. They are the most common and safest method for intra-hospital patient transport. During hospitalization, patients often need to be moved between departments—such as for diagnostics, consultations, or ward transfers—sometimes multiple times a day. This is especially critical for vulnerable individuals, including post-operative and immunocompromised patients. However, contaminated wheelchairs can pose a serious infection risk. Cleaning after each use is essential, yet time-consuming and not always thorough. To address this, we tested the effectiveness of the SAFE-HUG Wheelchair Cover, a disposable non-woven fabric that creates a protective barrier between the patient and the wheelchair. By limiting contact with potentially contaminated surfaces, it helps prevent cross-contamination. Our study evaluated its ability to block pathogens, focusing on *E. coli*.

Thus, these strategies are aligned with the growing need for sustainable and effective decontamination methods. They balance microbial control with environmental responsibility, offering promising solutions for the future of infection prevention in healthcare.

## 2. Material and Methods

### 2.1. Bacterial Cell Cultures

Bacterial strains were incubated for 24 h after thawing, under agitation, at 37 °C in specific culture media. In particular, *C. difficile* (NCTC 11204 strain; National Collection of Type Cultures, London, UK) was grown in Fluid Thioglycolate Medium (Merck, Rahway, NJ, USA) supplemented with 1% vitamin K1 (Fluka, Buchs, Switzerland), under anoxic conditions (Oxoid™ AnaeroJar™ and an Oxoid™ AnaeroGen™ Compact Sachet) (Oxoid Ltd., Basingstoke, UK). Meanwhile, *E. coli* (ATCC 25922D strain; American Type Culture Collection, Manassas, VA, USA) was cultured in LB medium, composed of 4% bactotryptone (Thermo Fisher Scientific, Waltham, MA, USA), 2% Bactoyeast extract (Thermo Fisher Scientific, Waltham, MA, USA), and 4% NaCl (Sigma-Aldrich Corporation, St. Louis, MO, USA). The final bacterial concentration used in the experiments was 10^6^ cells per 100 µL.

Both *C. difficile* and *E. coli* suspensions were subjected to cell counting using a Bürker chamber (Paul Marienfeld GmbH & Co. KG, Lauda-Königshofen, Germany) after 24 h of incubation. The counting was performed under an optical microscope at 40× magnification. Each bacterial suspension was then diluted with its respective culture broth to achieve a final concentration used for the experiments.

### 2.2. Preparation of Bacterial and RAMS Solution

The RAMS (Representative Artificial Mucosal Substrate) solution used in this study was adapted from standardized test soils described in the context of ISO/TS 15883-5 [25]. This technical specification outlines microbiological test procedures for evaluating the cleaning efficacy of mechanical processes applied to medical devices [26].

The RAMS solution was prepared by dissolving each powdered component in Phosphate-Buffered Saline (PBS) (Merck KGaA, Darmstadt, Germany) at the following concentrations: 0.6% bovine serum albumin (BSA) (Sigma-Aldrich Corporation, St. Louis, MO, USA; Catalog No. A7030), 1% mucin from bovine submaxillary glands (Sigma-Aldrich Corporation, St. Louis, MO, USA; Catalog No. M3895), and 3% maize starch (Commercially available food-grade maize starch was used in this study). Subsequently, bacterial suspensions were diluted 1:1 with the RAMS solution, resulting in a final bacterial concentration of 10^7^ cells/mL. The mixture was then homogenized by vortexing to ensure uniform distribution.

### 2.3. Contamination of Pans, Drying of Spots, Control Mix Solution Collection

Before contamination, the steel pans (stainless steel trays commonly used in clinical settings for the collection of fecal samples and biological fluids) were cleaned using a washer-disinfector. The cleaning program consisted of a wash phase with an alkaline detergent at 65 °C, followed by a neutralization phase with acid, and finally, a thermal disinfection phase at 93 °C for 1 min (A_0_ = 600). After this process, the pans underwent an additional cleaning step using 90% ethanol to ensure thorough decontamination.

Then, after, the pans were contaminated by placing eight spots of bacterial suspension, mixed with RAMS, on each pan. The spots were strategically positioned, with six spots on the border and two spots in the center of each pan. Each spot had a volume of 100 µL, resulting in a concentration of 10^6^ cells per spot. In each cycle, two pans were treated simultaneously, and the procedure was repeated at least three times to ensure sufficient data for statistical analysis.

The pans were then placed in a static incubator at 37 °C for 30 min to allow the spots to dry, ensuring the bacterial material adhered to the surface. Following this, the deposited material from half of the spots was collected using environmental sampling swabs (Neogen^®^ Quick Swab) (Neogen Corporation, Lansing, MI, USA; Manufacturer Part No. 700002007). The pans were subsequently introduced in the SMEG Bedpan Washer, and the remaining spots were collected after this washing process.

### 2.4. SMEG BPW1260 Bedpan Washer

The bedpan washing cycle consisted of several phases. Firstly, a pre-wash phase with cold water was carried out for two cycles, followed by a pre-wash phase with hot water. Then, a washing phase was performed using hot water and detergent BPW1260 (10 mL/L) and repeated four times. After that, a rinsing phase with hot water was performed three times, followed by a rinsing phase with hot water and limescale remover. Finally, the cycle concluded with a thermal disinfection phase according to the A_0_ parameter.

Importantly, the SMEG Bedpan Washer BPW, offers several wash programs— short, medium, and intensive—each comprising different washing phases and thermal disinfection phases with different A_0_ values set:

$$A_{0}=t\times 10^{\left(\frac{T-80}{10}\right)}$$

The longer the duration in seconds, the more effective the thermal disinfection process is t = time (s); T = temperature (°C).

This device unit is capable of achieving a maximum thermal disinfection A_0_ value of 6000. Thus, tests were performed at A_0__600 or A_0_ = 6000. The program used to verify the effectiveness of the bacteria reduction is Pr 08—IdProg. 708 (a specific program dedicated to thermal disinfection of sanitary bedpans and urine bottles. Energy consumption during the disinfection cycles was monitored using a calibrated power meter connected to the disinfection equipment. The meter recorded the total electrical energy (in kWh) consumed throughout each treatment cycle. Measurements started at the initiation of the cycle and ended upon completion, ensuring an accurate assessment of energy usage for both A_0_6000 and A_0_600 programs. This allowed comparison of energy efficiency between treatments under consistent operational conditions.

### 2.5. Analysis of the Bacterial Population After Disinfection

The efficiency of the disinfection method was tested by analyzing the bacteria present in the system following upon disinfection. Bacterial population was determined by several methods:

#### 2.5.1. Bacterial Colonies Count (CFU/mL)

After treatment, bacteria were recovered from the surfaces by vortexing each test surface in 10 mL of sterile phosphate-buffered saline (PBS) (Thermo Fisher Scientific Inc., Waltham, MA, USA), Catalog No. J61196.AP) for 1 min. Serial dilutions were plated on selective agar plates: Columbia Blood Agar (Thermo Scientific™, Thermo Fisher Scientific Inc.; Catalog No. CM0331T) for *C. difficile* (anaerobic incubation at 37 °C for 48 h) and MacConkey Agar (Thermo Scientific™ Oxoid™ Waltham, MA, USA; Catalog No. CM0007B) for *E. coli* (aerobic incubation at 37 °C for 24 h). Colony-forming units (CFU) were counted and expressed as CFU/mL.

#### 2.5.2. Bacterial Analysis by Optical Density (OD600 nm)

The optical density (OD600 nm) of the bacterial suspension was measured using a spectrophotometer, (Eppendorf Biophotometer #6131 (Eppendorf AG, 22,331 Hamburg, Germany) equipped with appropriate cuvettes. The measurements were performed both in the control samples (prior to thermal disinfection) to establish baseline absorbance values in the absence of thermal treatment, and after the thermal disinfection process to evaluate any potential changes in bacterial concentration. For each measurement, the bacterial suspensions were placed in clear cuvettes, and absorbance was recorded at a wavelength of 600 nm. This approach allowed for the comparison of bacterial suspension densities before and after disinfection, providing valuable insight into the effects of thermal treatment on bacterial viability and any reduction in bacterial concentration due to the disinfection process.

#### 2.5.3. Cell Count by Counting Chamber

For direct cell counting, bacterial samples were first stained with trypan blue dye (Trypan Blue Solution 0.4%, Thermo Fisher Scientific, Cat. No. 15250061) to differentiate viable from non-viable cells. The stained samples were then loaded into a counting chamber containing grids of known volume for manual enumeration. The chamber allows for the visualization and manual counting of individual bacterial cells within the grid area. The bacterial sample was diluted (1:100) prior to counting to ensure that the cells are sufficiently spaced for accurate enumeration. Once placed in the chamber, the sample was observed under a microscope (at magnification of 40×), and the number of cells within a defined grid area is counted.

#### 2.5.4. DNA Quantification

DNA was isolated from the samples using a column-based extraction procedure by using Qiagen DNA Kit (Cat. No./ID: 51104, Hilden, Germany) following the manufacturer specifications. The DNA concentration was quantified using a Qubit fluorometer and expressed in ng/µL, with the OD260 measurement serving as the standard for quantification.

### 2.6. Eco-Friendly Detergent Formulation

The eco-friendly detergent, based on sulfonated methyl esters, was prepared from waste sunflower cooking oil. The oil was first filtered and then subjected to transesterification and sulfonation to formulate the detergent. Due to the high variability of waste cooking oil samples, several trials were conducted by varying temperature, methanol-to-oil molar ratio, and reaction times. Additionally, we slightly modified the Resi Levi Permadani et al. protocol [27] by optimizing the amount of KOH, used as a basic catalyst, to 1.3% *w*/*w* based on the oil weight. Waste cooking oil (WCO) was initially heated to 60 °C, 70 °C, or 80 °C depending on the experimental setup. Methanol was used to produce methyl esters in different molar ratios (1:6, 1:9, 1:12). The catalyst was dissolved in methanol and heated to 30 °C before being added to the preheated oil. After different reaction times (30, 60, and 120 min), the mixture was cooled to allow phase separation between the trans-esterified product and the glycerol by-product. The glycerol was then removed using a separatory funnel. A further washing step with water at 30 °C was performed to remove excess soap. The resulting product was analyzed by GC-MS to verify the formation of methyl esters. Following confirmation of methyl ester presence, the sulfonation reaction was carried out by adding 1% (*w*/*v*) NaHSO_3_ to the esterified product. The reaction was conducted at 80 °C for 2 h under stirring (8000 rpm). Finally, the presence of sulfonated methyl esters was verified using FTIR spectroscopy. The formulated detergent was then employed for nanofluid preparation and subsequent testing.

An ISQ 7000 single quadrupole GC-MS system (Thermo Scientific™, Waltham, MA, USA) equipped with a capillary column (HP Innowax, 30 m × 0.25 mm (I.D) × 0.25 μm) was used to analyze the composition of the esterified product. Helium was used as the carrier gas, and about 1 μL of the solution was injected into the column. The injection temperature was 260 °C, and the oven started at a temperature of 40 °C, which was increased to 195 °C at a rate of 80 °C/min, before finally being raised to 225 °C at a rate of 5 °C/min and held for 60 min. Samples were diluted 1:1000 with hexane before analysis. The chemical structure of the MES detergent was characterized using Attenuated Total Reflectance (ATR) Fourier Transform Infrared (FTIR) spectroscopy, performed with the Thermo Scientific™ Nicolet™ iS50 spectrometer (Thermo Scientific™, Waltham, MA, USA). Spectral data were collected over the range of 4000–400 cm^−1^, with up to 128 scans acquired to improve signal quality and resolution. The ATR configuration enabled direct analysis of the liquid sample, offering an efficient approach for identifying the functional groups present in the eco-friendly detergent.

To evaluate the antimicrobial efficacy of the methyl-ester sulphonate detergent, *E. coli* cells (10^7^ cells/mL) were plated on Petri dishes and subjected to washing treatments. The washing procedure consisted of three consecutive rinses with either distilled water or the detergent solution, each followed by gentle agitation to ensure proper contact. Following treatment, bacterial load was quantified by direct cell counting using trypan blue staining and by DNA quantification, as previously described.

### 2.7. Nanofluid Formulation

Biomimetic magnetic nanoparticles and magnetic nanoparticles were synthesized and conjugated with silver salts to add antimicrobial activity to the formulated eco-friendly detergent, resulting in a nanofluid with both detergent and bactericidal properties.

MNPs and MamC-mediated BMNPs used in this study were synthesized according to Jabalera et al. [28]. Briefly, MamC cloning, expression, and purification were carried out as described by Peigneux et al. [29,30], and MamC was purified under denaturing conditions and then dialyzed to remove urea to allow the protein to correctly refold. Magnetite precipitation in the presence of MamC (BMNPs) or in the absence of any protein (MNPs) was carried out for 1 month in a closed system at 25 °C and 1 atm total pressure inside an anaerobic chamber (COY chamber) to avoid possible oxidation. The precipitates were recovered with a permanent magnet and washed with deoxygenated water.

Nanoassemblies of Ag electrostatically bound to BMNPs (Ag-BMNPs) or to MNPs (Ag-MNPs) were produced by taking advantage of the negative charge of both nanoparticles (isoelectric point (pI) of BMNPs is 4.4, and of MNPs is 7 [31]) when resuspended in HEPES buffer (pH = 7.4). To immobilize the silver ions on both surfaces, 5 mg of BMNPs or MNPs [resuspended and disaggregated in HEPES buffer (50 mM, pH 7.4)] were mixed with a Ag_2_SO_4_ solution containing 4 mg of Ag_2_SO_4_ in HEPES buffer. The silver ions, displaying a positive charge, interacted with both nanoparticles through electrostatic affinity. The nanoassemblies were left for 24 h at room temperature under rotation and were recovered with a permanent magnet. Three cycles of washing the nanoassemblies with the same HEPES buffer following their magnetic recovery were performed, and the nanoassemblies were used for the further experiments. The binding of the silver ion to the nanoparticles was verified by comparing the ζ-potential of BMNP, Ag-BMNPs, MNPs, and Ag-MNPs at pH 7.4 by using dynamic light scattering (DLS) with the Nano ZetaSizer ZS ZEN3600 (Malvern Instruments, Malvern, Worcestershire, UK) [32]. The size was also investigated for both naked and silver-coupled particles using nanoparticle tracking analysis (NTA) with a NanoSight NS300 (Malvern Panalytical, Malvern, UK). Samples were sonicated for 5 min with a bath sonicator and then diluted 50-fold in Milli-Q water before analysis. Three 60 s runs (1498 frames total) were performed at camera level 11 and a detection threshold of 3–5. Hydrodynamic diameters and particle concentrations (particles/mL) were automatically calculated from the Brownian motion of the particles using the Stokes–Einstein equation [33] as reported here below.

$$R_{h}=\frac{k_{B}T}{6\pi \eta D}$$

being R_h_ the hydrodynamic radius (m), k_B_ the Boltzmann Constant (≈1.38 × 10^−23^  J/K), T the temperature (K), η the solvent viscosity (kg/m s), and D the diffusion coefficient (m^2^/s).

### 2.8. Antibacterial Activity of the Methyl Ester and Nanofluid Compounds

The methyl-ester sulphonate detergent, as well as the silver nanoparticles, were tested either alone or in combination with each other on 30 *E. coli* strains obtained from patients or wastewater sources (Supplemental Table S1). The detergent was diluted with PBS to a final 10% concentration. Ag-BMNPs and Ag-MNPs, at initial concentration of 5 mg/mL, and after a washing step with PBS, were diluted 1:10 with PBS or with the 10% concentrated detergent. The effects of the detergent and the nanoparticles were assessed through minimum inhibitory concentration (MIC) and minimum bactericidal concentration (MBC) tests. MIC_50_ and MIC_90_ represent the minimum inhibitory concentrations required to inhibit the growth of 50% and 90% of the tested bacterial strains, respectively. Similarly, MBC_50_ and MBC_90_ indicate the minimum bactericidal concentrations required to kill 50% and 90% of the strains. Bacterial suspensions were adjusted to a density of 0.5 McFarland (corresponding to 10^8^ cells/mL) and then diluted 1:10 with Mueller–Hinton broth (MBH; Thermo Scientific™ Oxoid™, Waltham, MA, USA; catalog No. CM0405B). MIC was performed in 96-well plates, putting in each well 75 µL of MBH mixed—in the first column of each plate—with 75 µL of either the 10% detergent, the nanoparticles diluted in PBS, or the nanoparticles diluted with the detergent. Then, 1:2 serial dilutions of these mixes were performed in the plates. In each well, 75 µL of bacterial dilution was then added. The MIC was assessed after 24 h incubation at 37 °C by checking bacterial growth in each well.

MBC was performed with plating on agar Petri dishes with 5 µL of the content of each well of the MIC test plates, after 24 h of incubation, in which bacterial growth was not observed; the first well in which growth was observed for each bacterial strain was also tested. MBC was then assessed after 24 h of incubation at 37 °C.

### 2.9. Wheelchair Cover

The SAFE-HUG PRO Wheelchair Cover was provided by EASYLINKED S.r.l. (20122 Milan, Italy). The commercial version of the cover used in this study has been tested and certified according to ISO 22610:2006 [34] and EN 14126:2004 [35], which evaluate the resistance of protective textiles to bacterial penetration under moist and mechanical conditions. To assess its barrier effectiveness against microbial penetration, a controlled in vitro test was performed using *E. coli* as the test organism under growth-promoting conditions within an incubator (incubation at 37 °C), aiming to mimic a worst-case scenario for bacterial proliferation. A sample of the cover was placed in direct contact with a total of 5 × 10^7^ cells of *E. coli* on a sterile Petri dish. After 1 h and 24 h of incubation, the side of the cover that had not been in direct contact with the bacteria was analyzed to determine if any microorganisms had passed through the material. Thus, the three complementary methods above detailed were employed to assess bacterial penetration: Optical Density (OD600) measurements to estimate bacterial growth, cell counts to quantify viable bacterial cells, and DNA quantification to detect and measure the presence of bacterial genetic material.

### 2.10. Statistical Analysis

Data were analyzed using Student’s *t*-test to compare the differences between the control and experimental groups. The *t*-test was applied to assess whether there were significant differences in the measured values, such as bacterial concentration or absorbance, between samples before and after thermal disinfection. All statistical analyses were performed using SPSS for Windows, version 22.0 (SPSS Inc., Chicago, IL, USA, 2013). A *p*-value < 0.05 was considered statistically significant. The results are presented as the mean ± standard deviation (SD) of at least three independent experiments.

## 3. Results

### 3.1. Efficacy of the SMEG BPW1260 Bedpan Washer-Disinfector in Reducing Bacterial Contamination

**Figure 1:**
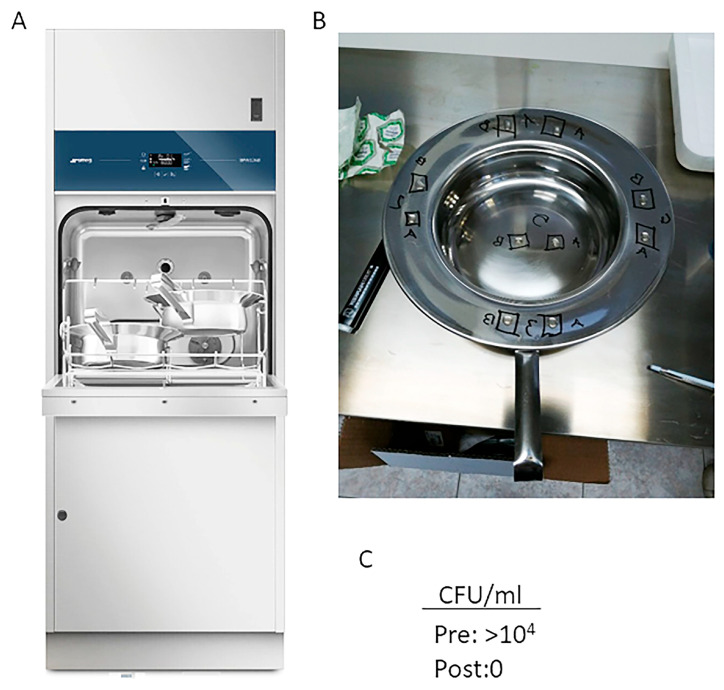
(**A**) SMEG BPW1260 bedpan washer-disinfector. (**B**) The bedpan image showing 5 of the 10 spots corresponding to the Pre value ((**A**): before disinfector cycles) and the remaining 5 spots corresponding to the Post value ((**B**): after thermal disinfection). (**C**) CFU/CFU/mL before (PRE) and after (POST) thermal disinfection.

To assess the ability of the thermodisinfector (Figure 1A) to reduce bacterial contamination on bedpans, we initially contaminated specific spots on the pans with a mixture of RAMS and bacterial cells. In particular, the bacterial/RAMS mixture was applied to four spots (duplicate) along the border and one spot (duplicate) at the center of each bedpan (Figure 1B). The bedpans were then dried at 37 °C. After drying, five of the ten spots corresponding to the Pre value (A: before disinfector cycles) were collected, serving as the baseline samples (i.e., the amount of contamination prior to treatment). The remaining five spots were collected after thermal disinfection with SMEG Bedpan Washer. To evaluate *C. difficile* reduction after thermal disinfection, we employed the Colony Forming Unit (CFU/CFU/mL) method, along with spectrophotometry, cell counting, and DNA quantification. The evaluations were performed at least three times in two different pans. Using the A06000 program of the thermodisinfector, we did not observe any colony growth of *Clostridium difficile*. Therefore, we further investigated whether thermal disinfection could also be effective with the A0600 program, which would consume less energy. Therefore, as shown in Figure 1C, no *Clostridium difficile* colonies grew after thermal disinfection.

**Figure 2:**
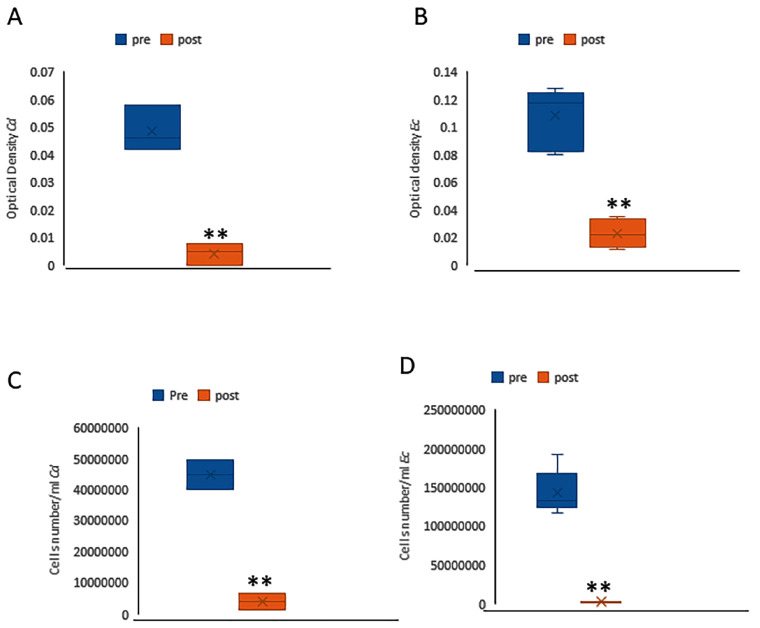
Graph shows the absorbance values before (blue) and after (orange) thermal disinfection for *Clostridium difficile* (**A**) and *Escherichia coli* (**B**). This decrease in bacterial population was also confirmed by the bacterial counts obtained through the counting chamber method for *Clostridium difficile* (**C**) and *Escherichia coli* (**D**). ** (*p* < 0.005).

Thus, we assessed the ability of the thermal disinfection to reduce the presence of *C. difficile* as well as *E. coli* by using spectrophotometry, cell counting, and DNA quantification after thermal disinfection. The absorbance (OD600 nm) of *C. difficile* and *E. coli* suspensions was measured using a spectrophotometer in the samples before and after thermal disinfection, with each bacterial strain analyzed separately. In addition to absorbance measurements, bacterial counts were performed using a counting chamber to determine the bacterial concentration in the samples. As shown in Figure 2, a significant reduction in absorbance (Figure 2A,B) and a decrease in bacterial cell count (Figure 2B,C) were observed for both bacterial strains after thermal disinfection.

**Figure 3:**
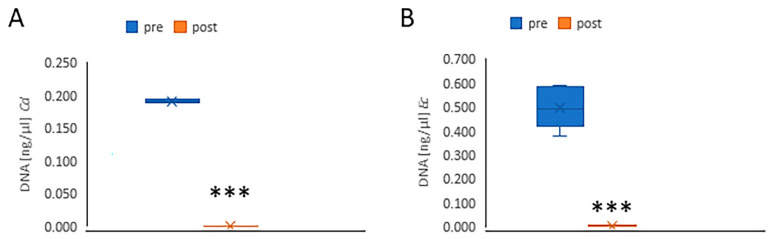
The graph shows the concentration of extracted DNA before (blue) and after (orange) thermal disinfection. The reduction in DNA levels following thermal disinfection was statistically significant for both *Clostridium difficile* (**A**) and *Escherichia coli* (**B**). *** *p* < 0.0005.

The amount of DNA present in the bacterial samples was quantified by measuring the optical density (OD) at 260 nm, which is commonly used to determine nucleic acid concentration. The results were expressed as DNA concentration (ng/µL). Measurements were taken both before and after thermal disinfection to assess any potential reduction in DNA levels due to the treatment and washing. A significant reduction in DNA levels was observed following thermal disinfection for both *C. difficile* and *E coli* strains (Figure 3), indicating that the treatment effectively compromised the bacterial DNA. These findings are consistent with the absorbance and bacterial count data, collectively providing strong evidence of the effectiveness of thermal disinfection in decreasing bacterial viability and genetic material.

### 3.2. Energy Efficiency and Bacterial Load Reduction

The results obtained with the A_0_6000 and A_0_600 treatments (as previously reported) both demonstrated a significant reduction in bacterial load, indicating the effectiveness of both parameters in the disinfection process. As shown in the table below (Table 1), the A_0_600 treatment resulted in a considerable improvement in energy efficiency compared to A_0_6000. Despite achieving effective disinfection, the A_0_600 cycle consumed only 0.42 kWh of energy, compared to the 0.77 kWh required by the A_0_6000 cycle (that means that there is a reduction of 45% in energy consumption), with a reduction in cycle time (28 min versus 35 min). This improvement in energy efficiency highlights the superior performance of the A_0_600 treatment without compromising disinfection effectiveness.

### 3.3. Synthesis of the Methyl Esters

The chemical process under investigation entails the conversion of waste sunflower cooking oil into a biodegradable detergent via a two-step reaction: transesterification followed by sulfonation. This approach is noteworthy not only for its sustainable utilization of waste materials but also for its alignment with the increasing demand for environmentally friendly cleaning agents. During transesterification, triglycerides derived from waste sunflower cooking oil (SWCO) react with methanol in the presence of a potassium hydroxide catalyst, yielding fatty acid methyl esters and glycerol as a by-product [36]. Sulfonation is a chemical reaction that introduces a sulfonic acid group (–SO_3_H) into an organic compound, typically using reagents such as sulfur trioxide (SO_3_) or oleum—a mixture of sulfur trioxide and sulfuric acid [37]. In this study, sodium bisulfite (NaHSO_3_) was employed as the sulfonating agent due to its milder reactivity, which allows for greater control over the reaction and reduces the risk of over-sulfonation or unwanted side reactions. This makes it particularly suitable for modifying sensitive organic compounds such as fatty acids. The sulfonation process increases the polarity and water solubility of the resulting molecules, properties that are especially beneficial for applications in detergents and surfactants.

**Figure 4:**
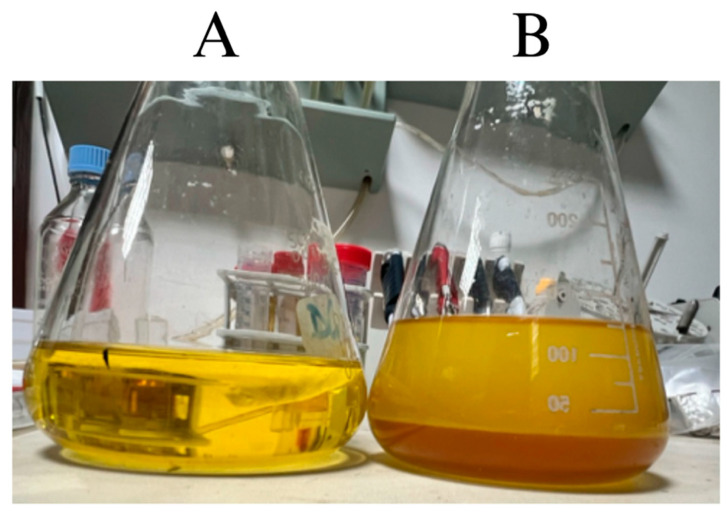
Waste cooking oil pre- (**A**) and post-transesterification (**B**) where phase separation between esters (yellow) and glycerol (brown) was observed.

**Figure 5:**
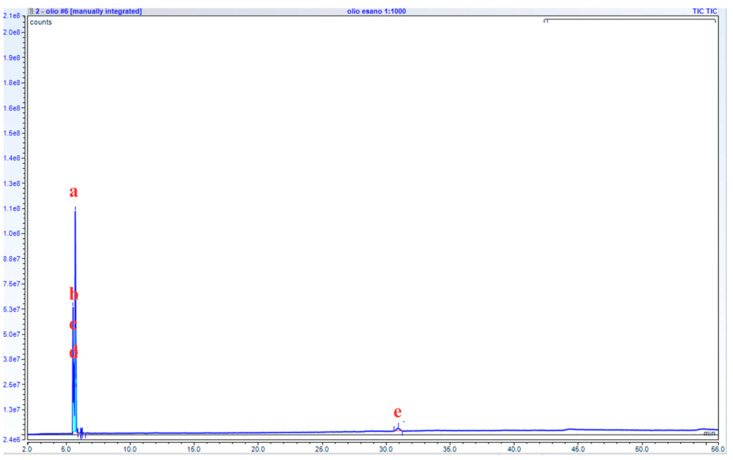
GC-MS chromatogram of waste cooking oil before being subjected to the transesterification process. (2-Propanone, methylhydrazone (a), 1-Methyl-2-propenylhydrazine (b), Azetidine (c), 2-Ethylthiirane (d), Ethyl tetracosanoate (e)).

During the transesterification process, two phases appeared in the reaction vessel: one containing the esterified compounds and the other containing the non-useful mixture (Figure 4). As shown in Figure 5, the filtered waste cooking oil did not exhibit ester groups. The GC-MS chromatogram reveals the presence of various volatile and semi-volatile compounds characteristic of thermo-oxidative degradation products formed during repeated use of sunflower oil for cooking: Peak a corresponds to 2-Propanone, methylhydrazone, a derivative of acetone—a common secondary product of lipid peroxidation and β-scission of oxidized unsaturated fatty acids; peaks b (1-methyl-2-propenylhydrazine) and c (Azetidine) are small nitrogen-containing heterocycles typically formed through reactions between reactive carbonyl compounds (e.g., aldehydes and ketones) generated during triglyceride breakdown and amino compounds originating from food materials such as batters, meat, or vegetable proteins. Their presence is therefore indicative of used, rather than fresh, cooking oil; peak d is identified as 2-Ethylthiirane, a sulfur-containing volatile compound likely produced via thermal degradation of sulfur amino acids such as cysteine or methionine, commonly found in fried foods; and peak e, Ethyl tetracosanoate, is a long-chain waxy fatty acid ethyl ester (C_24_), formed through fatty acid esterification or ester–exchange reactions involving high-molecular-weight components under prolonged heating conditions [38,39].

Among the various trials conducted, the reaction performed at 60 °C for 30 min with a methanol-to-oil molar ratio of 1:9 showed the best phase separation between the ester products and the glycerol by-product, suggesting complete transesterification, when the basic catalyst was increased to 1.3% *w*/*w*. These results are comparable to those reported by Resi Levi Permadani et al. [27].

**Figure 6:**
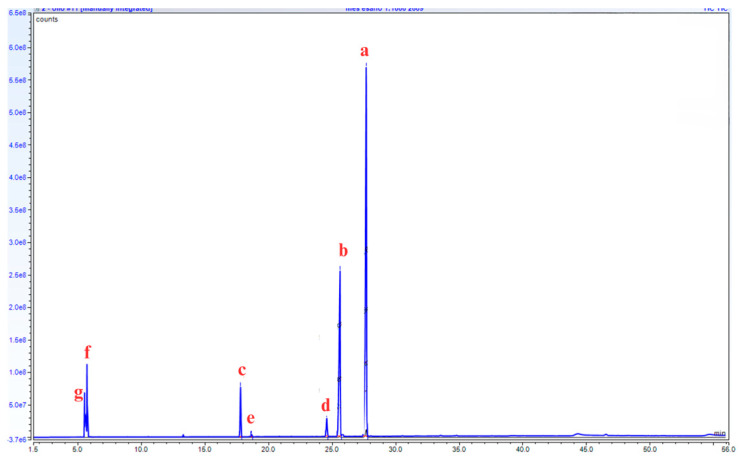
GC-MS chromatogram of waste cooking oil after the transesterification reaction, showing the detection of 5 different methyl ester compounds (9,12-Octadecadienoic acid (Z,Z)-methyl ester (a), 9-octadecenoic acid (Z)-methyl ester (b), hexadecanoic acid methyl ester (c), methyl stearate (d), 9-hexadecenoic acid methyl ester (e)), and the presence of residual 2-propanone, methylhydrazone (f), and azetidine (g).

The chromatogram in Figure 6 shows the presence of several methyl esters, with 9,12-Octadecadienoic acid (Z,Z)-methyl ester as the major component (63.2%), and 28.2% of 9-octadecenoic acid (Z)-methyl ester. The complete methyl ester composition is shown in Table 2.

### 3.4. Methyl Ester Sulfonation

**Figure 7:**
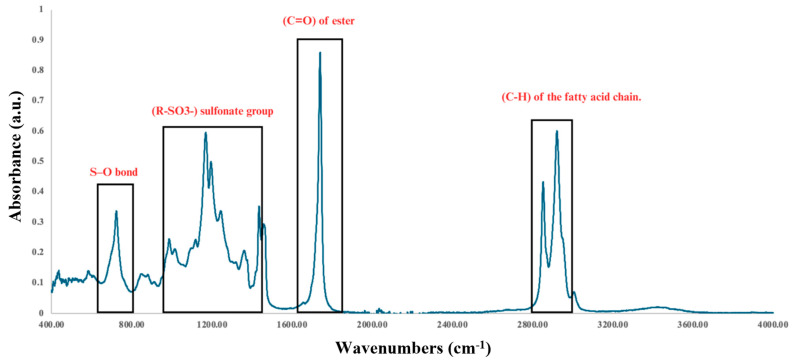
FTIR spectrum of the methyl ester sulfonate showing both sulfonate groups (R-SO_3_^−^) and ester (C=O) groups, as well as sulfonic (S-O) and fatty acid chain (C-H).

The product obtained after sulfonation was analyzed by FTIR to determine its composition (Figure 7). Two peaks at 2853 and 2924 cm^−1^ identify the aliphatic groups of the fatty acid chains. The presence of a strong peak at 1740 cm^−1^ indicates the carbonyl (C=O) group of the [27,40,41].

The presence of absorption bands in the 1015–1361 cm^−1^ region is typical of the stretching vibrations of sulfonate groups (R-SO_3_^−^) [27,41].

**Figure 8:**
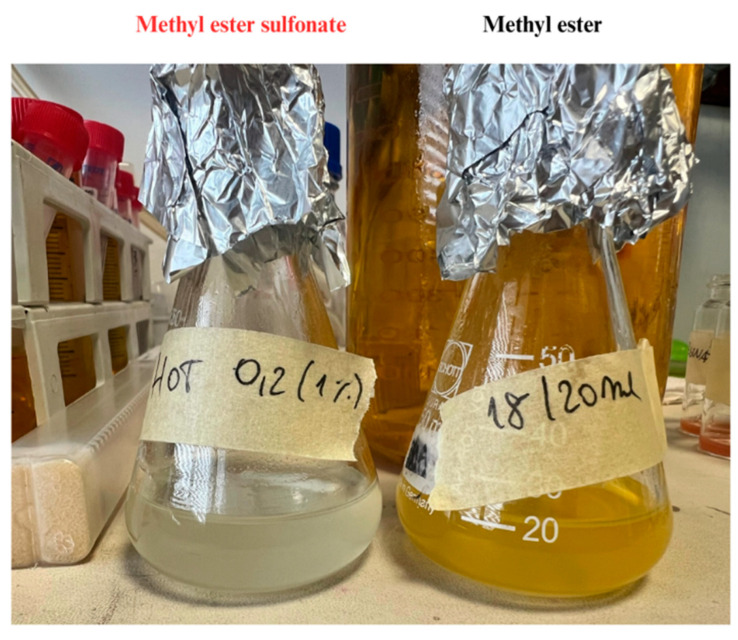
The transparent product indicates the methyl ester sulfonate (**left flask**), while the methyl ester before sulfonation appears yellow (**right flask**).

According to the FTIR spectrum, the methyl esters were sulfonated, confirming the successful preparation of a transparent MES-based detergent (Figure 8). Furthermore, no characteristic band was observed around 3500 cm^−1^, indicating the absence of alcoholic or water residues.

Thus, to evaluate the antimicrobial efficacy of the methyl-ester sulphonate detergent, *E. coli* cells were plated on Petri dishes and subjected to washing treatments using distilled water or the detergent solution. Bacterial load was subsequently quantified. The data (Table 3) show a significant reduction in bacterial load after treatment with the methyl-ester sulphonate detergent, with no detectable viable cells or bacterial DNA (*p* < 0.001). Washing with distilled water also led to a significant reduction in cell counts and DNA levels, although to a lesser extent (*p* < 0.01 and *p* < 0.05, respectively). These results confirm the superior efficacy of the detergent in reducing microbial contamination.

### 3.5. Particle’s Characterization

The immobilization of silver ions was performed on two different types of nanoparticles, BMNPs and MNPs, taking advantage of the pI and establishing an electrostatic bond between the surface of the nanoparticles and the silver cation. ζ-potential values after the coupling put in evidence changes in the surface of the nanoassemblies compared to that of unloaded BMNPs or unloaded MNPs, confirming the successful presence of silver ions. ζ-potential values for Ag-BMNPs and Ag-MNPs are close to or above zero in HEPES 50 mM at pH 7.4, while unloaded nanoparticles in the same buffer showed a higher density of negative charges on the surface, such as for BMNPs (−32.00 ± 2.10 mV) and for MNPs (−27.00 ± 3.00 mV) (Table 4). This change for nanoassemblies confirms the presence of silver ions on the surface, as negatively charged groups (carboxyl or hydroxyl groups) [31] previously present at the nanoparticle surfaces are now blocked by silver cations.

**Figure 9:**
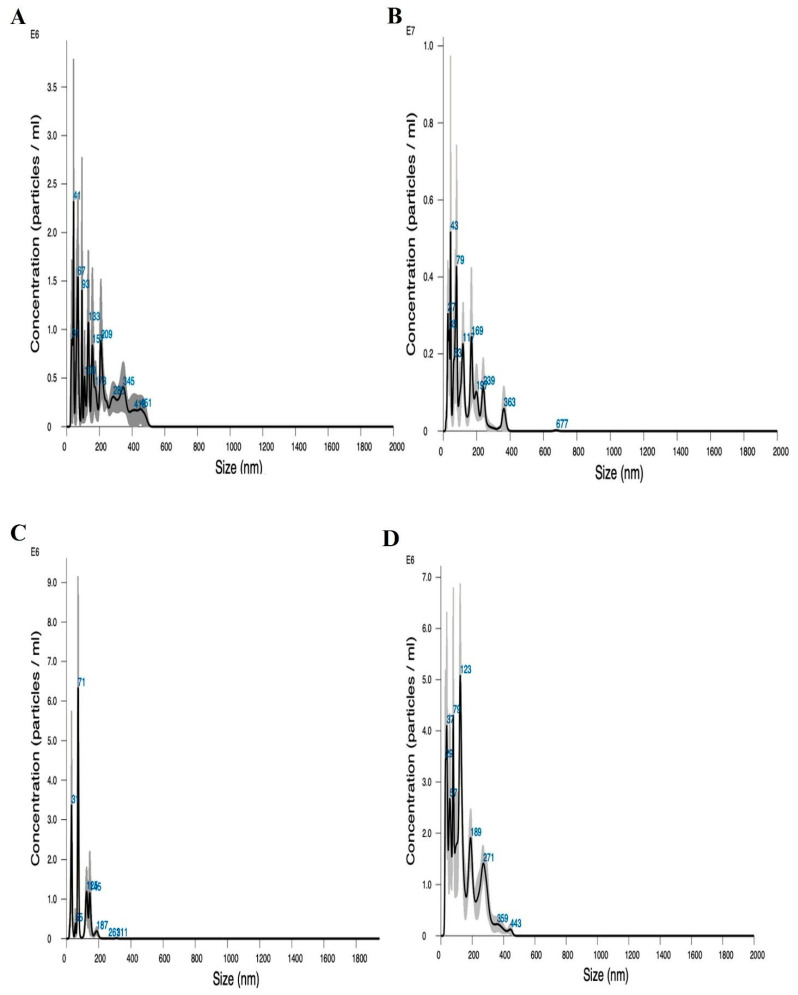
NTA of (**A**) naked MNPs, (**B**) MNPs coupled with silver, (**C**) naked BMNPs, and (**D**) BMNPs coupled with silver.

NTA of bare magnetic nanoparticles suspended in 50 mM HEPES buffer at pH 7.4 revealed a heterogeneous size distribution ranging from 41 nm to 93 nm, with a particle concentration of (8.87 ± 1.82) × 10^7^ particles/mL (Figure 9A). These findings are consistent with the dynamic light scattering (DLS) data reported by Donini et al. [42]. Upon coupling MNPs with silver, two distinct particle populations emerged at approximately 43 nm and 79 nm, with a concentration of (2.43 ± 0.11) × 10^8^ particles/mL (Figure 9B). In contrast, bare biomimetic magnetic nanoparticles exhibited a more homogeneous distribution centered around 71 nm, with a concentration of (6.08 ± 0.75) × 10^7^ particles/mL (Figure 9C), in agreement with previous findings [38]. When BMNPs were functionalized with silver, a heterogeneous profile was observed, characterized by two predominant peaks at approximately 123 nm and 79 nm, and a particle concentration of (1.77 ± 0.44) × 10^8^ particles/mL (Figure 9D).

### 3.6. Antibacterial Ability of the Methyl-Ester Sulphonate Detergent Combined with Silver-Derivatized Magnetic Nanoparticles

The methyl-ester sulphonate detergent, the two types of silver magnetic nanoassemblies, and a mixture of the detergent and the nanoassemblies were tested against 30 *E. coli* strains. To assess their antibacterial potential, we evaluated the minimum inhibitory concentration (MIC) and the minimum bactericidal concentration (MBC).

The results, expressed as mean values of the tested strains, demonstrated that BM-treated *E. coli* had a MIC50 of 0.063 (±0.0067) mg/mL and a MIC90 of 0.119 (±0.0067) mg/mL. In comparison, MN-treated *E. coli* had a MIC50 of 0.031 (±0.0049) mg/mL and a MIC90 of 0.063 (±0.0049) mg/mL. Notably, the MBC50 values were identical for both BM and MN, at 0.063 (±0.0075) mg/mL, while the MBC90 values were 0.125 (±0.0075) mg/mL for both types of nanoparticles. Although no direct cell counting was performed after treatment, the absence of visible regrowth on agar plates was interpreted as an indication of bactericidal activity, in line with standard microbiological definitions. These findings suggest that both MN and BM exhibit comparable growth inhibition and bactericidal activity on *E. coli* strains tested.

### 3.7. Barrier Performance Results of the SAFE-HUG PRO Wheelchair Cover

The barrier effectiveness of the SAFE-HUG PRO Wheelchair Cover was assessed through a controlled in vitro test using *E. coli* as the test organism. First, we assessed the potential microbial presence on the covers. No detectable microbial load was observed, as indicated by the absence of optical density (OD) signals, cell counts, and DNA levels. Thus, we evaluated the cover’s performance under growth-promoting conditions within an incubator. After placing a sample of the cover in direct contact with a high bacterial load (5 × 10^7^ cell number), the opposite (non-exposed) side was analyzed after 1 h and 24 h in incubators using three complementary methods: OD600 (optical density), cells count, and DNA quantification. The results show a substantial reduction in bacterial transmission compared to the uncovered control, demonstrating the material’s strong barrier properties (Table 5).

Thus, the observed reduction in cell concentrations after 1 h exceeds three orders of magnitude (>10^3^), while the reduction after 24 h corresponds with approximately three orders of magnitude.

## 4. Discussion

The growing challenge of antimicrobial resistance, along with the limitations of conventional chemical disinfectants, highlights the urgent need for innovative and sustainable infection control strategies in healthcare settings [43,44]. Thus, we focused on critical points to prevent contamination in hospital environments, proposing targeted solutions for each. In particular, we tested the effectiveness of a system for cleaning steel pans and developed an eco-friendly detergent as a strategy for effective disinfection, while the use of wheelchair covers serves as an additional protective barrier. This integrated approach allows intervention on multiple fronts of infection transmission, significantly contributing to risk reduction.

The SMEG BPW1260 proved to be a reliable and effective device for thermal disinfection, operating in accordance with the European Standard EN ISO 15883. Its ability to significantly reduce bacterial contamination suggests its suitability for applications requiring high hygiene standards, such as in healthcare and laboratory settings [45]. The observed reduction in DNA content further supports the notion of a true bactericidal effect, as it indicates not only the elimination of viable bacteria but also the degradation of their genetic material—an important aspect when considering the risk of residual pathogenicity or gene transfer [46]. Beyond microbiological efficacy, evaluating the operational performance of different disinfection cycles was essential to identify solutions that are both effective and energy-efficient. The Ao concept enables the calculation of all viable time–temperature combinations required to achieve a desired level of disinfection, optimizing both efficiency and cost-effectiveness according to the specific needs [47]. Among the tested programs, in our system, the A0600 cycle emerged as the most advantageous. It maintained a comparable level of bacterial inactivation while markedly reducing both energy consumption and cycle time. This dual benefit is particularly relevant in the context of sustainable practices and resource management, where reducing environmental impact without compromising safety is a growing priority [48].

The reduced consumption—from 0.77 kWh to 0.42 kWh—and shortened duration—from 35 to 28 min—demonstrate that optimization of disinfection protocols is possible and desirable. In high-throughput environments, such improvements can translate into lower operational costs and increased workflow efficiency [49]. Moreover, adopting energy-efficient cycles contributes to institutional sustainability goals, aligning with broader ecological and economic considerations [50,51]. In detail, this addresses the recent appeals from supranational bodies, including the International Hospital Federation and the Pan-European Commission on Health and Sustainable Development. They call on healthcare organizations to take greater responsibility for their negative environmental impact and develop green initiatives in order to reduce their energy consumption, a major contributor to their carbon footprint [52]. Additionally, as traditional microbiological methods may not detect non-culturable organisms and often require extended processing times [53], incorporating complementary validation strategies such as DNA quantification provides a more robust assessment of bactericidal efficacy [46,54]. In our study, the use of an integrated system—combining direct cell counting for viability assessment, optical density measurement, and/or DNA quantification—significantly enhanced the assessment of microbial contamination. Unlike traditional culture-based methods such as colony-forming unit (CFU) counts, which are time-intensive and unable to detect viable but non-culturable organisms, our approach enabled faster and more comprehensive microbial detection. This strategy not only reduced the time required for microbiological validation but also improved sensitivity and reliability, offering a more accurate and timely assessment of the bactericidal efficacy of disinfection procedures. This study also emphasizes the need for standardized biological validation protocols for BWDs, ensuring their performance can be reliably evaluated across different clinical contexts. A limitation of the present study is the focus on only two bacterial pathogens, *Escherichia coli* and *Clostridium difficile*. Although these microorganisms are clinically relevant and commonly associated with healthcare-associated infections, this limited scope may not fully represent the broad spectrum of pathogens encountered in healthcare settings. Future research should aim to evaluate the efficacy of the proposed treatments against a wider range of microorganisms, including multidrug-resistant strains, to enhance the applicability and robustness of the results.

In parallel, we assessed the antibacterial efficacy of a novel nanofluidic detergent formulated from waste cooking oil and enriched with silver-derivatized nanoparticles. This detergent represents a sustainable alternative to conventional chemical agents, contributing to circular economy goals by repurposing waste materials. This environmentally friendly detergent consisting of sulfonated methyl esters was effectively synthesized from waste cooking oil through a two-step process combining transesterification and sulfonation reactions. Indeed, as evidenced by FTIR spectroscopy, both ester and sulfonate groups are present. The esterification reaction resulted in a 92 ± 5% conversion of waste oil into methyl ester, while a complete transformation of the methyl ester into methyl ester sulfonate was observed, as evidenced by the color change from yellow to white. Moreover, the observed predominance of C18 methyl esters is of particular interest due to its positive correlation with enhanced detergency of methyl ester sulfonates [27].

Silver nanoparticles (AgNPs) have emerged as potent antimicrobial agents due to their broad-spectrum activity and unique physicochemical properties. Their nanoscale size allows for enhanced interaction with microbial surfaces, while the release of silver ions (Ag^+^) contributes to their bactericidal effects [55]. AgNPs have been shown to induce visible damage to the cell membranes of both *E. coli* and *C. difficile*, resulting in cell lysis and death. By compromising bacterial defenses, they enhance antimicrobial efficacy and allow for a reduction in the required dosage of conventional antibiotics [56,57]. In the recent past, silver-functionalized magnetite nanoparticles have demonstrated potent antibacterial activity, particularly against *E. coli*, through mechanisms involving reactive oxygen species generation and silver ion release, leading to membrane disruption and cell death. Although direct studies on *C. difficile* are limited, the known efficacy of AgNPs against Gram-positive anaerobes suggests that such nanoparticles may offer a promising strategy for targeting *C. difficile* as well [58,59,60]. The incorporation of Ag-MNPs and Ag-BMNPs into the sulfonated methyl ester-based detergent represents a promising strategy for enhancing antimicrobial performance in healthcare disinfection protocols. This study demonstrated that both types of nanoassemblies, when combined with this eco-friendly detergent derived from waste cooking oil, exhibit significant bactericidal activity against Escherichia coli strains.

The successful functionalization of MNPs and BMNPs with silver ions was confirmed by ζ-potential measurements, which revealed a marked shift from strongly negative values (−27.00 ± 3.00 mV for MNPs and −32.00 ± 2.10 mV for BMNPs) to values near neutrality or slightly positive after silver binding. This shift is indicative of electrostatic interactions between the negatively charged nanoparticle surfaces and the positively charged silver ions, resulting in surface charge neutralization. Nanoparticle tracking analysis further supported these findings, showing increased heterogeneity and size distribution in the silver-functionalized samples, particularly in Ag-BMNPs, suggesting the formation of larger or more complex nanoassemblies. Both nanoassemblies demonstrated comparable bactericidal efficacy, as reflected by similar MBC50 and MBC90 values.

The enhanced antimicrobial activity observed in these formulations likely results from a synergistic mechanism involving the membrane-disruptive properties of the MES detergent and the well-documented bactericidal effects of silver ions, which interfere with bacterial DNA replication and metabolic processes. Additionally, the magnetic properties of the nanoparticles offer potential advantages in terms of targeted application and paves the way for a post-use recovery, contributing to the sustainability of the disinfection system. Although the present study demonstrated the in vitro antimicrobial efficacy of the methyl-ester sulphonate detergent, further research is needed to validate its performance under real-world conditions. In particular, testing on contaminated hospital surfaces would provide more clinically relevant insights into its disinfection potential.

Overall, the integration of Ag-MNPs and Ag-BMNPs into a biodegradable, waste-derived detergent provides a dual benefit: improved antimicrobial efficacy and alignment with circular economy principles. Future studies should focus on evaluating the long-term stability of these nanoformulations and their effectiveness against a broader spectrum of pathogens, including multidrug-resistant organisms. Other promising disinfection approaches currently under investigation, which do not rely on nanotechnology and do not directly contribute to circular economy models, include contact-less methods such as the use of essential oils in gaseous form and gaseous ozone. Essential oils are natural, biodegradable, and leave no harmful residues, representing an environmentally friendly option [61]. Their antimicrobial activity is effective but can be variable depending on the oil composition and pathogen type [62]. Gaseous ozone is a powerful oxidizing agent that can rapidly disinfect surfaces without chemical residues, but it requires specialized equipment and safety precautions due to its toxicity at high concentrations [63].

In addition, this study highlights the critical role that wheelchairs can play in the transmission of healthcare-associated infections (HAIs), a risk that is often underestimated in routine infection control protocols [64]. Our findings demonstrate the effectiveness of the SAFE-HUG PRO Wheelchair Cover in minimizing bacterial transfer under conditions designed to simulate real-world contamination. Even following prolonged exposure to *E. coli*, the cover significantly prevented bacterial penetration to the non-contact surface. In contrast, unprotected wheelchair surfaces showed high bacterial loads and detectable levels of DNA, confirming their potential as reservoirs and vectors of microbial transmission. These results emphasize the importance of implementing physical barriers such as the covers as a practical and effective measure to enhance existing hygiene practices [64]. This approach is especially valuable in high-risk healthcare environments, where indirect transmission routes—such as contaminated mobility aids—are frequently overlooked. Incorporating such protective solutions into standard protocols may significantly reduce the microbial burden, contribute to patient safety, and align with broader goals of sustainability and infection prevention in healthcare settings.

## 5. Conclusions

The use of no-touch thermal disinfection, sustainable nanoparticle-enhanced detergent and wheelchair cover represents a powerful strategy for improving infection control in healthcare settings. The SMEG BPW1260 washer-disinfector demonstrated strong bactericidal activity against *C. difficile* and *E. coli*, with the A_0_600 cycle specifically providing increased energy efficiency without compromising effectiveness. Simultaneously, the novel biofluidic detergent showed enhanced antimicrobial properties, especially when combined with nanoparticles. In addition to surface and equipment disinfection, the use of protective barriers for high-contact medical devices such as wheelchairs plays a critical role in preventing microbial transmission. The SAFE-HUG PRO Wheelchair Cover proved to be a highly effective barrier against *E. coli* penetration. These findings underscore the importance of incorporating physical containment strategies—such as antimicrobial wheelchair covers—into broader infection prevention protocols.

Together, these technologies offer complementary benefits and, when integrated into routine procedures, have the potential to significantly reduce the burden of healthcare-associated infections (HAIs) while supporting the transition toward more sustainable and resilient healthcare practices. However, continued research is essential to optimize their application, ensure efficacy across a broader spectrum of pathogens, and evaluate their long-term impact in clinical environments.

## Figures and Tables

**Table 1:** Steam condensation, cooling, and energy consumption analysis.

			Steam Condensation and Cooling	400 V THREE-PHASE	
SHORT NAME	ID PROG	A_0_	T Target [°C]	ENERGY (KWh)	CYCLE TIME (min)
Pr 08	708	6000	55	0.77	35
Pr 08	708	600	55	0.42	28

**Table 2:** Methyl ester composition after transesterification, calculated from peak areas in the GC-MS chromatogram.

Methyl Ester Composition	Percentage (%)
9,12-octadecadienoic acid (Z,Z)	63.2
9-octadecenoic acid (Z)	28.2
hexadecanoic acid	5.7
methyl stearate	2.7
9-hexadecenoic acid (Z)	0.2

**Table 3:** Antimicrobial efficacy of the methyl-ester sulphonate detergent.

Analysis Method	Treatment	Pre-Treatment	Post-Treatment	Significance
Cell counting	Distilled water	(2.41 ± 0.84) × 10^7^ cells/mL	(3.84 ± 0.72) × 10^5^ cells/mL	*p* < 0.01
	Methyl-ester sulphonate detergent	(2.40 ± 0.71) × 10^7^ cells/mL	Not detectable	*p* < 0.001
DNA extraction	Distilled water	0.272 ± 0.35 ng/µL	0.140 ± 0.04 ng/µL	*p* < 0.05
	Methyl-ester sulphonate detergent	(7 ± 1.35) × 10^4^ ng/µL	Not detectable	*p* < 0.001

**Table 4:** ζ-potential outcomes for bare and silver ion-conjugated nanoparticles (MNPS and BMNPs). All the measurements have been performed in triplicate.

Sample	ζ-Potential (mV)
MNPS	−27.00 ± 3.00
Ag-MNPS	−0.21 ± 3.00
BMNPS	−32.00 ± 2.10
Ag-BMNPs	+0.11 ± 0.02

**Table 5:** Barrier Performance.

Analysis Method	With Cover (1 h)	Control (1 h)	With Cover (24 h)	Control (24 h)
Optical density (OD600)	0.000	0.024 ± 0.0012	0.000	0.100 ± 0.009
Cells/mL	(7 ± 1.35) × 10^4^	(1.6 ± 0.112) × 10^8^	(1 ± 0.87) × 10^5^	(2.36 ± 0.713) × 10^8^
DNA (ng/µL)	Not detectable	0.2 ± 0.014	Not detectable	0.6 ± 0.054

## Data Availability

The original contributions presented in this study are included in the article/Supplementary Material. Further inquiries can be directed to the corresponding authors.

## Supplementary Materials

The following supporting information can be downloaded at: https://www.mdpi.com/article/10.3390/microorganisms13081923/s1, Table S1: List of *E. coli* strains used in this study.
