# Supplementary material for: Evaluation of Biocontrol Measures to Reduce Bacterial Load and Healthcare-Associated Infections
Source: Microorganisms. 2025 Aug 18;13(8):1923. doi: 10.3390/microorganisms13081923 (PMC12388564; doi:10.3390/microorganisms13081923)
Supplement: Supplementary file 1 [file microorganisms-13-01923-s001.zip › microorganisms-3796116-supplementary.pdf]

Supplemental Table S1. List of E. coli strains used in this study

Strain Code	Source of Isolation	Pathogenicity / Virulence Data
19044CE5	Wastewater	Not determined
19042CE4	Wastewater	Not determined
1948OE3	Wastewater	Not determined
19029OE1	Wastewater	Not determined
19034EE1	Wastewater	Not determined
19040CE1	Wastewater	Not determined
19040OE1	Wastewater	Not determined
19044OE5	Wastewater	Not determined
19049EE3	Wastewater	Not determined
20074CE6	Wastewater	Not determined
20026OE1	Wastewater	Not determined
20015OE1	Wastewater	Not determined
19074OE5	Wastewater	Not determined
19074CE5	Wastewater	Not determined
20025CE6	Wastewater	Not determined
20010CE2	Wastewater	Not determined
20074CE1	Wastewater	Not determined
19052EE2	Wastewater	Not determined
19051EE1	Wastewater	Not determined
20025CE1	Wastewater	Not determined
20014OE2	Wastewater	Not determined
19078OE3	Wastewater	Not determined
URO 1	Patient	Not determined
URO 3	Patient	Not determined
URO 4	Patient	Not determined
URO 5	Patient	Not determined
URO 6	Patient	Not determined
URO 17	Patient	Not determined
URO 22	Patient	Not determined
URO 23	Patient	Not determined
